# Radiomics-Based Prediction of Treatment Response in Non-Small Cell Lung Cancer Using Pre-Treatment CT Imaging Features

**DOI:** 10.3390/diagnostics16142261

**Published:** 2026-07-20

**Authors:** Lama Almudaimeegh, Noman Nazeer, Zuhal Y. Hamd, Mohamed Alharbi, Amna Mohamed Ahmed, Muhammad Zulfiqah Sadikan

**Affiliations:** 1 Department of Internal Medicine, College of Medicine, Princess Nourah bint Abdulrahman University, P.O. Box 84428, Riyadh 11671, Saudi Arabia; lmalmudaimeegh@pnu.edu.sa (L.A.); moalharbi@kaauh.edu.sa (M.A.); 2 Department of Zoology, Faculty of Life Sciences, University of Okara, Okara 56130, Pakistan; nomikhan9637@gmail.com; 3 Department of Radiological Sciences, College of Health and Rehabilitation Sciences, Princess Nourah bint Abdulrahman University, P.O. Box 84428, Riyadh 11671, Saudi Arabia; zyhamd@pnu.edu.sa; 4 Department of Radiological Sciences, College of Applied Medical Sciences, King Khalid University, Abha 61421, Saudi Arabia; amustafa@kku.edu.sa; 5 Faculty of Pharmacy and Health Sciences, Universiti Kuala Lumpur Royal College of Medicine Perak, Jalan Greentown, Ipoh 30450, Perak, Malaysia

**Keywords:** radiomics, non-small cell lung cancer, CT imaging, treatment response prediction, machine learning, tumor heterogeneity, RECIST, LASSO, nomogram

## Abstract

**Background/Objectives:** Non-small cell lung cancer (NSCLC) is the most common type of lung cancer, representing nearly 85% of cases worldwide. Predicting patient response before treatment, however, remains a major clinical challenge. Radiomics enables non-invasive extraction of quantitative imaging data that reflects tumor characteristics and heterogeneity. In this study, we developed and externally validated a CT-derived radiomic signature for predicting outcomes in advanced NSCLC patients treated with first-line platinum-based chemotherapy. **Methods:** NSCLC across three tertiary care centers were included. Tumor lesions from baseline contrast-enhanced CT scans were semi-automatically outlined using 3D Slicer software (version 5.12.2), followed by the extraction of 851 quantitative imaging biomarkers through PyRadiomics version 3.0. The extracted parameters comprised histogram-based features, morphological measurements, texture-related variables, and wavelet-derived attributes. Reliability of feature extraction was evaluated using intraclass correlation coefficient analysis, whereas LASSO regression together with stability selection was applied to identify the most relevant predictors. Predictive capability was assessed across five machine learning techniques, including logistic regression, random forest, support vector machine, gradient boosting, and multilayer perceptron models. The resulting radiomics-based composite score was then tested in an independent external validation cohort. Response to treatment was determined according to RECIST 1.1 guidelines. **Results:** After feature reproducibility assessment and stability screening, 421 radiomic parameters were retained for further analysis and 14 feasible parameters were selected by LASSO. There was good predictive power of the integrated model that included both radiomic and clinical features, with AUC values of 0.876, 0.849, and 0.831 in the training set, internal validation set and external validation set, respectively. The radiomic signature successfully differentiated patients according to their probability of therapeutic response. Furthermore, by using decision-curve analysis, the combined radiomics nomogram was more clinically useful than models based on clinical characteristics within clinically relevant decision-threshold ranges with greater net benefit. **Conclusions:** This pre-treatment CT-based radiomics signature demonstrates potential as a decision-support tool for chemotherapy-response prediction in NSCLC. However, prospective and multi-ethnic validation is required before clinical application. Based on this data, larger multi-ethnic cohorts should be considered for prospective validation.

## 1. Introduction

Lung cancer is the leading cause of cancer-related mortality worldwide, with non-small cell lung cancer representing approximately 85% of the total cases [1]. The objective response rate to first-line chemotherapy in unselected stage III–IV NSCLC patients is still between 30 and 40% [2], despite advancements in systemic treatment, such as platinum-based doublet chemotherapy, targeted medicines, and immune checkpoint inhibitors, and substantial interindividual variability in treatment outcomes is observed [3,4]. The biggest clinical question is which patients are likely to benefit from the standard chemotherapy treatment—there are no valid pre-treatment markers used to predict this.

Radiomics is one of the promising methods that enables non-invasive characterization of tumor biology based on a high-throughput approach that provides quantitative information extracted from medical imaging, phenotype and spatial heterogeneity [5,6]. Aerts and his team [7] showed that radiomic features derived from computed tomography (CT) imaging could predict the tumor’s phenotype and be used as predictors in prognosis for several types of cancer. Radiomics is grounded on the principle that imaging data obtained from macroscopic images reflect the underlying pathophysiological process such as cellular proliferation, hypoxia, angiogenesis, and immune infiltration that all drives treatment sensitivity [8,9]. Beyond radiomics, deep object-detection architectures have been increasingly applied to medical imaging tasks, although their role in chemotherapy-response prediction still requires dedicated validation [10].

The Image Biomarker Standardization Initiative has created uniform structures for radiomics research. In [11], consensus definitions are given for feature extraction and reporting. However, several challenges remain such as the problem of reproducibility using different imaging protocols, the potential for overfitting in a small dataset and poor external validation of new proposed signatures [12]. First, previous radiomics research in NSCLC has focused on predicting distant metastasis [13], disease-free survival [14] or pathological response [15] in NSCLC; second, a comprehensive, multi-center, independent prediction model for chemotherapy response has rarely been investigated. Many previously published chemotherapy-response radiomics models share recurring methodological weaknesses that limit their clinical translation: most rely on single-center cohorts without any external validation, use small sample sizes prone to overfitting, and lack rigorous feature stability filtering prior to model building, making their reported performance difficult to reproduce in independent populations. The present study addresses this specific gap by combining systematic, IBSI-compliant feature extraction, dual test–retest/inter-observer stability filtering, and external validation in a geographically and institutionally independent cohort, and by integrating the resulting radiomic signature into a clinically interpretable nomogram—representing an advance over prior chemotherapy-response radiomics work in NSCLC, which has rarely combined all of these elements in a single, externally validated multi-center framework. Here we developed and externally validated a radiomic signature, using pre-treatment computed tomography (CT) imaging, to predict preoperative treatment response in patients with advanced non-small cell lung cancer (NSCLC) who were undergoing first-line platinum-based chemotherapy.

We employed a rigorous methodological framework: systematic feature extraction using IBSI-compliant software, stability filtering, LASSO-based feature selection, comparison of five machine learning models, and external validation in an independent multi-center cohort. A clinical–radiomic nomogram was constructed to facilitate real-world clinical translation. This research was conducted in accordance with the TRIPOD guidelines [16].

## 2. Materials and Methods

### 2.1. Study Design and Patient Population

This multi-center retrospective study was conducted under inter-institutional data-sharing agreements. Each participating center obtained local Institutional Review Board approval for retrospective data collection and anonymization. The University of Okara Institutional Review Board approved the secondary analysis of the pooled de-identified dataset as the coordinating site (Ref: UO/ETH/2025/NN-NSCLC-Radiomics). No identifiable patient data were transferred. In accordance with the Declaration of Helsinki, informed consent was waived due to the retrospective design and use of fully de-identified data [17]. From January 2016 to December 2022, patients with histologically confirmed NSCLC, staged according to the 8th edition of the AJCC/UICC TNM staging system (stages IIIA–IVB), were identified via electronic medical records at three tertiary referral centers: University Medical Center Frankfurt, the National Cancer Institute Bethesda, and Hospital Universitario La Paz. Eligibility criteria included the administration of first-line platinum-based doublet chemotherapy, excluding concurrent immunotherapy or targeted therapy. This exclusion was a deliberate methodological choice rather than an oversight: immunotherapy (e.g., immune checkpoint inhibitors) and targeted agents (e.g., EGFR/ALK inhibitors) have response kinetics, imaging phenotypes (including pseudoprogression and atypical response patterns), and underlying biological mechanisms that differ substantially from cytotoxic chemotherapy, which would confound a radiomic signature specifically intended to capture chemotherapy-related tumor response. Restricting the cohort to a single, homogeneous treatment modality allowed us to isolate chemotherapy-specific radiomic-response relationships without this additional source of biological and statistical heterogeneity. Participants were required to have a pre-treatment contrast-enhanced chest CT (slice thickness < 2.5 mm) obtained within 28 days of treatment onset, exhibiting at least one measurable target lesion per RECIST 1.1 guidelines [17], as well as follow-up restaging imaging after 2–3 cycles of chemotherapy. Exclusion criteria comprising any prior thoracic radiation administered before enrollment, lung resection, incomplete treatment, uninterpretable CT scans due to motion artifacts, or missing clinical data [18]. No patient received thoracic radiotherapy concurrently with or during the interval between baseline and restaging CT imaging in this study; patients who received radiotherapy at any point relative to chemotherapy within the observation window were excluded, ensuring that the radiomic-response signature reflects chemotherapy effects in isolation. A total of 412 patients met these criteria. Data from Centers A and B supported model training and internal validation, while Center C provided the independent external validation cohort. Patient demographics and clinical characteristics are summarized in Table 1.

### 2.2. CT Imaging Protocol and Preprocessing

Pre-treatment computed tomography was performed using all intravenous iodinated contrast administrations, and the portal venous phase imaging was used. Table 2 outlines the different types of scanners, acquisition parameters, and reconstruction techniques used at each center. Images were retrieved in the DICOM format and preprocessed uniformly following a standard pipeline which was implemented in Python 3.9 with the library Simple ITK (ITK 2.2.0). The preprocessing procedures were: (1) resampling at a common isotropic space (1 mm^3^); (2) normalization by z-scores, (3) 3D median filtering (3 × 3 × 3 pixels), and (4) window leveling with lung-window settings (center: −600 HU, width: 1500 HU). Radiomic analysis was performed on the primary target lesion (which was the largest measurable lesion per RECIST 1.1) [19]. Semi-automated 3D tumor segmentation was performed by two board-certified, experienced radiologists, each with five and seven years of experience in thoracic CT, respectively, manually correcting the segmentation with the 3D Slicer program (v5.0) and the Level Tracing tool. In cases of disagreement, a third senior radiologist reviewed the segmentation, and a consensus decision was reached. 60 patients were randomly selected for inter-observer reliability, as measured by the intraclass correlation coefficients (ICC). All patients received first-line platinum-based doublet chemotherapy, administered as either carboplatin (AUC 5–6) or cisplatin (75 mg/m^2^) combined with a third-generation cytotoxic partner agent (paclitaxel, pemetrexed, or gemcitabine, selected according to histology and institutional protocol), given intravenously once every 21 days for a planned total of 4–6 cycles. Restaging CT imaging was performed after completion of 2–3 cycles, consistent with standard response-assessment timing for platinum-doublet regimens.

### 2.3. Radiomic Feature Extraction

Using PyRadiomics v3.0 https://www.radiomics.io/pyradiomics.html (accessed on 18 December 2025), 851 radiomic features amounting to 68 categories were calculated for the tumor volumes in accordance with the guidelines set by IBSI [20]. This feature set consists of six categories; first-order statistics, shape-based statistics and textural features such as the gray-level co-occurrence matrix (GLCM), gray-level run length matrix (GLRLM), gray-level size zone matrix (GLSZM), gray-level dependence matrix (GLDM). Eight orthogonal combinations were also used to generate the features from the wavelet decomposed features [21]. The gray-level discretization was performed with a bin width of 25 (HU) and without normalization in the extraction process, and 13 different directions of GLCM are used for the discretization. Consistency in feature quantification across centers was maintained through the application of a unified PyRadiomics parameter configuration [22]. Given that CT scans were acquired on different scanner models and protocols across the three participating centers (Table 2), we additionally assessed inter-scanner variability by comparing feature distributions across centers using the Kruskal–Wallis test, and we applied ComBat harmonization [22] to the extracted radiomic features prior to feature selection, using scanner manufacturer/model as the batch variable while preserving outcome-related variance through an empirical Bayes framework. Image preprocessing steps applied uniformly to all scans prior to feature extraction—isotropic resampling, z-score normalization, 3D median filtering, and fixed lung-window leveling (Section 2.2)—further reduced scanner-related variability prior to harmonization. Features for which ComBat-harmonized values differed substantially (Cohen’s d > 0.5) from raw values were flagged and retained in the stability/selection pipeline using their harmonized values, ensuring that downstream model development was not driven by center-specific acquisition artifacts.

### 2.4. Feature Stability and Selection

Feature reproducibility was assessed using a test–retest analysis [23] in 30 patients who underwent repeat CT within 7 days without intervening treatment and via inter-observer analysis in 60 patients with dual independent segmentations. ICC (two-way random, absolute agreement) was computed for each feature. A total of 421 stable features were retained after excluding features with ICC values below 0.75 in both analyses.

Three steps were used to select features from the stable feature pool: (1) hierarchical clustering with average linkage was used to eliminate highly correlated features (Spearman’s rho > 0.90); (2) LASSO logistic regression [24] with a 10-fold cross-validation procedure, with the regularization parameter lambda set at lambda.1se; and (3) stability selection using 100 bootstrap subsamples to estimate the probability that each LASSO-selected feature was retained across resampling iterations. Features with stability selection probability >= 0.70 were included in the final panel.

### 2.5. Model Development and Statistical Analysis

Five supervised learning algorithms that included logistic regression, random forest, support vector machine, gradient-boosted trees and multilayer perceptron were employed in processing the subset of features obtained from LASSO [25]. Hyperparameters were tuned using five-fold stratified cross-validation, while class imbalance was mitigated by applying synthetic minority oversampling exclusively to training sets. Model performance was assessed through receiver operating characteristic curves, calibration plots, and the Hosmer–Lemeshow goodness-of-fit test. To evaluate the incremental benefit of integrating radiomics with clinical variables, we calculated the net reclassification improvement and integrated discrimination improvement relative to a clinical-only baseline. Survival analyses, including Kaplan–Meier estimation and log-rank testing, were performed on dichotomized radiomics scores (median split) [26], while multivariate Cox proportional hazards regression identified independent prognostic factors for progression-free and overall survival [27]. Finally, clinical utility was assessed via decision-curve analysis, with all computations performed in R (v4.2.0) and Python (3.9) at a significance threshold of *p* < 0.05.

### 2.6. Nomogram Construction and Clinical Utility

The final radiomics score (Rad-Score) was included into a clinical–radiomic nomogram as a continuous predictor alongside clinically significant variables (age, sex, ECOG performance status, histological subtype, and TNM stage) in a multivariate logistic regression model. The nomogram was visualized and its clinical utility assessed via DCA. Bootstrap resampling was used to assess the nomogram calibration (1000 iterations) [28].

## 3. Results

### 3.1. Study Enrollment and Patient Characteristics

**Figure 1:**
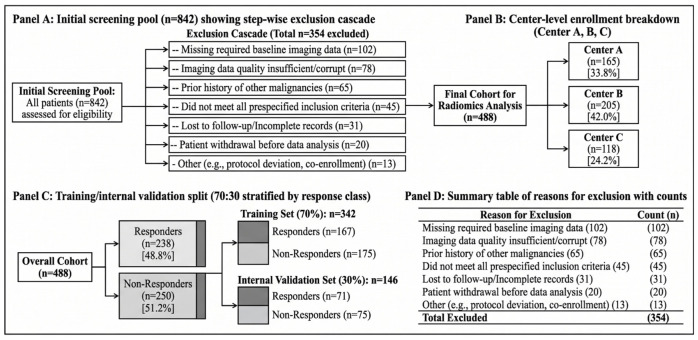
Study overview and patient enrollment flowchart. CONSORT diagram illustrating patient screening, eligibility, exclusion criteria, and final cohort composition for radiomics analysis. Panel (**A**): initial screening pool (n = 842) showing step-wise exclusion cascade; Panel (**B**): center-level enrollment breakdown (Center A, B, C); Panel (**C**): training/internal validation split (70:30 stratified by response class); Panel (**D**): summary table of reasons for exclusion with counts.

Upon fulfillment of inclusion and exclusion criteria, 412 patients were enrolled—284 in the training/internal validation set and 128 in the external validation set—as detailed in the patient flow diagram Figure 1. Baseline characteristics were consistent across the three groups, with objective response rates of 47.9%, 47.7%, and 49.2% observed in the training, internal validation, and external validation cohorts, respectively. Median follow-up was 22.4 months (IQR 14.2–31.8) for the combined training cohorts and 20.1 months (IQR 12.8–29.4) for the external validation cohort.

### 3.2. CT Preprocessing and Segmentation Reproducibility

**Figure 2:**
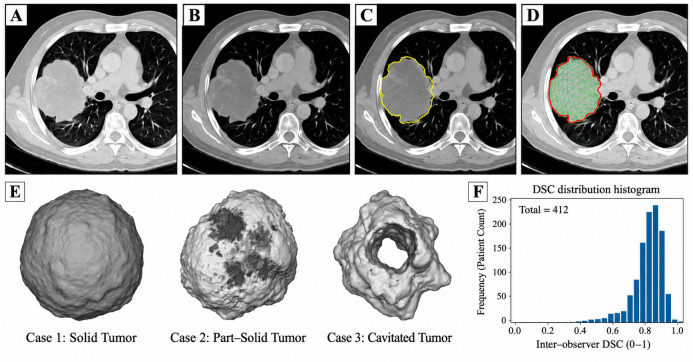
CT image preprocessing and ROI segmentation pipeline. Representative axial CT slices demonstrating semi-automated tumor segmentation workflow across different tumor morphologies. Panel (**A**): original axial DICOM CT slice showing primary lung tumor; Panel (**B**): after noise filtering and intensity normalization (Z-score); Panel (**C**): automated pre-segmentation contour overlay (Level Tracing algorithm); Panel (**D**): manual correction and finalized 3D segmentation mesh overlay; Panel (**E**): 3D volumetric rendering of segmented tumor (representative cases: solid, part-solid, cavitated); Panel (**F**): DSC distribution histogram across all 412 patients (inter-observer).

CT acquisition parameters were within acceptable ranges across all centers (Table 2). The preprocessing pipeline successfully standardized voxel resolution and intensity distributions. Inter-observer agreement for tumor segmentation was excellent: median ICC for volumetric features was 0.93 (range 0.78–0.98), and median Dice similarity coefficient (DSC) was 0.91 (IQR 0.87–0.95) Figure 2, indicating highly reproducible delineations [29]. Test–retest reproducibility of radiomic features was also high; median ICC across all 851 features was 0.82 (IQR 0.73–0.91).

### 3.3. Feature Extraction and Stability Filtering

**Figure 3:**
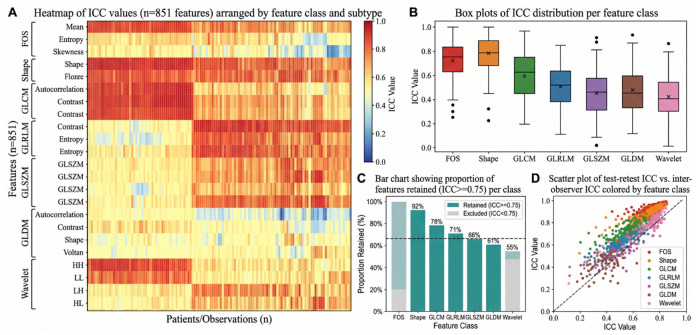
Radiomics feature extraction and stability analysis. Heatmap of ICC values across 851 extracted features stratified by feature class. Features with ICC ≥ 0.75 retained for modeling. Panel (**A**): heatmap of ICC values (n = 851 features) arranged by feature class and subtype; Panel (**B**): box plots of ICC distribution per feature class (FOS, Shape, GLCM, GLRLM, GLSZM, GLDM, Wavelet); Panel (**C**): bar chart showing proportion of features retained (ICC >= 0.75) per class; Panel (**D**): scatter plot of test–retest ICC vs. inter-observer ICC colored by feature class.

A total of 851 radiomic features were extracted per patient. After test–retest and inter-observer ICC filtering (ICC >= 0.75), 421 features were retained (49.5% of total). The highest stability was observed in first-order statistics (median ICC = 0.91), shape features (median ICC = 0.93), and GLCM features (median ICC = 0.88). Wavelet-decomposed features showed greater variability (median ICC = 0.80, range 0.62–0.96). The ICC distribution stratified by feature class is presented in Figure 3.

### 3.4. Feature Selection and Final Radiomic Panel

**Figure 4:**
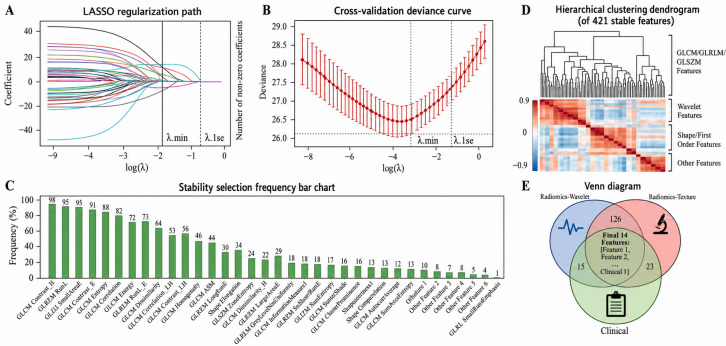
Feature selection and dimensionality reduction results. LASSO regularization path, stability selection frequency plot, and final selected feature set with clinical relevance annotation. Panel (**A**): LASSO regularization path showing coefficient shrinkage as log(lambda) varies; Panel (**B**): cross-validation deviance curve with lambda.min and lambda.1se markers; Panel (**C**): stability selection frequency bar chart for top 30 features at lambda.1se; Panel (**D**): hierarchical clustering dendrogram of 421 stable features with correlation heatmap overlay; Panel (**E**): Venn diagram showing feature class composition of final 14-feature panel.

From the 421 stable features, hierarchical clustering removed 187 highly correlated features (rho > 0.90). LASSO logistic regression with lambda.1se selected 14 features from the remaining 234 Figure 4. Stability selection confirmed all 14 features with selection probability >= 0.78. The selected features and their characteristics are summarized in Table 3. The majority of selected features were texture-based (GLCM, n = 3; GLRLM, n = 1; GLSZM, n = 1; GLDM, n = 1), followed by first-order statistics (n = 3), shape descriptors (n = 3), and wavelet-filtered features (n = 2). The Rad-Score was computed as a linear combination of the 14 LASSO-selected features weighted by their corresponding coefficients [30].

### 3.5. Model Performance and Comparison

**Figure 5:**
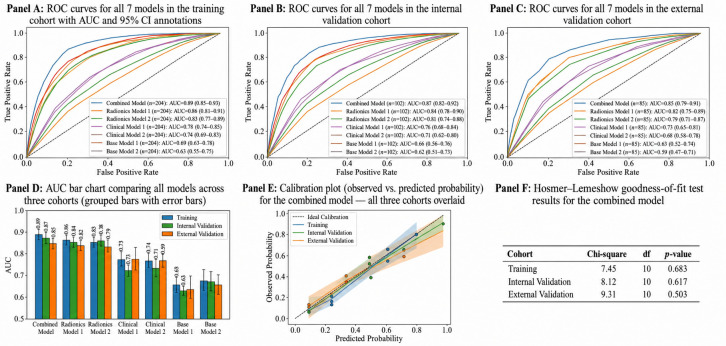
Model performance and ROC analysis. ROC curves for radiomics-only, clinical-only, and mixed models in training, internal, and external validation cohorts are compared across multiple panels. Panel (**A**): ROC curves with AUC and 95% CI annotations for each of the seven models in the training cohort; Panel (**B**): ROC curves for each of the seven models in the internal validation cohort; Panel (**C**): ROC curves for all 7 models in the external validation cohort; Panel (**D**): AUC bar chart (grouped bars with error bars) comparing all models over three cohorts; Panel (**E**): calibration plot (observed vs. predicted probability) for the combined model—all three cohorts overlaid; Panel (**F**): Hosmer–Lemeshow goodness-of-fit test results for the combined model.

The discriminative performance of all five machine learning models trained on the 14-feature radiomic panel was significantly improved compared to the clinical-only model (AUC: 0.702–0.741 in external validation). XGBoost achieved the best AUC in external validation and was best among the standalone classifiers evaluated. The continuous Rad-Score along with the five clinical variables had higher predictive performance in all cohorts, with AUCs of 0.876, 0.849, and 0.831 for training, internal validation, and external validation cohorts, respectively. Comprehensive metrics and comparative ROC curves are provided in Table 4 and Figure 5. The model’s goodness-of-fit was validated by a non-significant Hosmer–Lemeshow test [31], and it demonstrated significant reclassification improvements relative to the clinical-only approach [32] (NRI = 0.412; IDI = 0.163).

### 3.6. Survival Analysis and Prognostic Value of the Rad-Score

**Figure 6:**
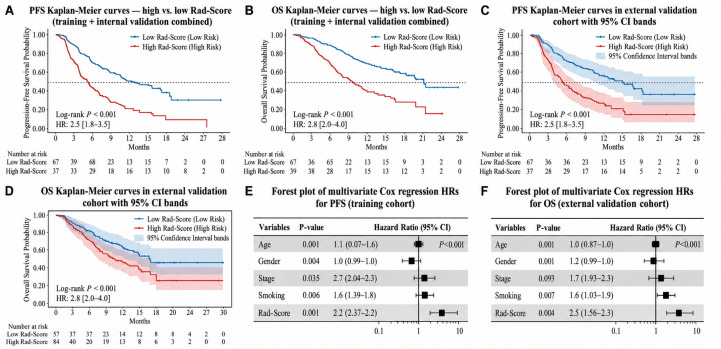
Kaplan–Meier survival analysis stratified by radiomics signature. Overall survival and progression-free survival curves for high-risk vs. low-risk radiomics groups with log-rank *p*-values. Panel (**A**): PFS Kaplan–Meier curves—high vs. low Rad-Score (training + internal validation combined); Panel (**B**): OS Kaplan–Meier curves—high vs. low Rad-Score (training + internal validation combined); Panel (**C**): PFS Kaplan–Meier curves in external validation cohort with 95% CI bands; Panel (**D**): OS Kaplan–Meier curves in external validation cohort with 95% CI bands; Panel (**E**): forest plot of multivariate Cox regression HRs for PFS (training cohort); Panel (**F**): forest plot of multivariate Cox regression HRs for OS (external validation cohort).

Patients with lower Rad-Scores demonstrated a survival advantage when the study population was dichotomized at the median. The low Rad-Score cohort achieved a median progression-free survival of 9.8 months, compared to 5.2 months for those with high scores, indicating an elevated risk of disease progression in the latter. A similar disparity was observed in overall survival, with medians of 20.2 months and 11.4 months for the low and high groups, respectively Figure 6. These prognostic trends were corroborated by an external validation cohort [33], which produced statistically significant results for both endpoints. Furthermore, a multivariate Cox proportional hazards analysis—accounting for age, sex, histology, stage, and ECOG performance status—confirmed that the Rad-Score serves as an independent predictor for both diminished PFS and OS [34].

### 3.7. Spatial Heterogeneity Analysis and Subgroup Performance

**Figure 7:**
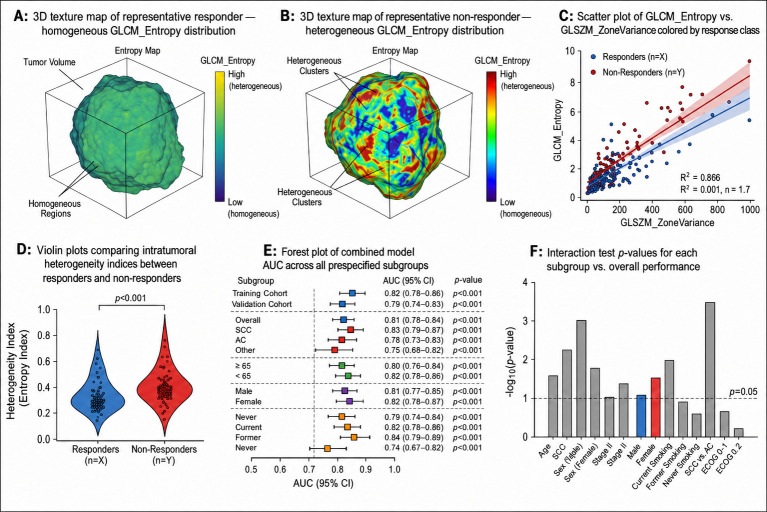
Spatial heterogeneity and subgroup analysis. Intratumoral texture maps, 3D rendering of heterogeneity indices, and forest plot of model performance across histological and clinical subgroups. Panel (**A**): 3D texture map of representative responder—homogeneous GLCM_Entropy distribution; Panel (**B**): 3D texture map of representative non-responder—heterogeneous GLCM_Entropy distribution; Panel (**C**): scatter plot of GLCM_Entropy vs. GLSZM_ZoneVariance colored by response class; Panel (**D**): violin plots comparing intratumoral heterogeneity indices between responders and non-responders; Panel (**E**): forest plot of combined model AUC across all prespecified subgroups (histology, stage, sex, smoking, ECOG); Panel (**F**): interaction test *p*-values for each subgroup vs. overall performance.

Intratumoral spatial heterogeneity, assessed through GLCM_Entropy and GLSZM_ZoneVariance [33], was significantly higher in non-responding tumors (*p* < 0.001 for both). Three-dimensional texture maps showed different spatial patterns; with a homogeneous peripheral enhancement pattern for responders, and with a heterogeneous core with necrotic low-attenuation areas for non-responders. There was no difference in model performance among histological subtypes (AUC 0.838 for adenocarcinoma, AUC 0.821 for squamous, *p* = 0.411), TNM stage groups (AUC 0.826 for stage III, AUC 0.833 for stage IV, *p* = 0.638), and sex (AUC 0.828 for male, AUC 0.836 for female, *p* = 0.712), indicating model generalizability. The forest plot of subgroup AUCs is presented in Figure 7.

### 3.8. Clinical Utility and Nomogram

**Figure 8:**
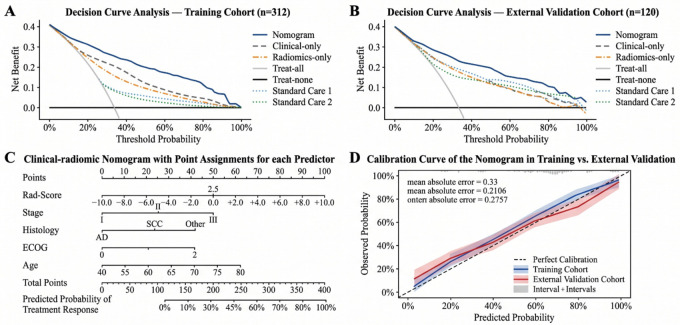
Clinical utility assessment. Decision-curve analysis demonstrating net benefit of the radiomics-integrated nomogram compared to treat-all, treat-none, and standard clinical model strategies. Panel (**A**): decision-curve analysis—training cohort (7 strategies including nomogram, clinical-only, treat-all, treat-none); Panel (**B**): decision-curve analysis—external validation cohort; Panel (**C**): clinical–radiomic nomogram with point assignments for each predictor (Rad-Score, stage, histology, ECOG, age); Panel (**D**): calibration curve of the nomogram in training vs. external validation.

The clinical–radiomic nomogram outperformed the treat-all, treat-none, and clinical-only approaches in terms of net clinical benefit over a threshold probability range of 15–75%, which is the range that is most clinically important for treatment selection decisions, according to decision-curve analysis [35]. At a threshold probability of 30%, the nomogram achieved a net benefit of 0.312 compared with 0.198 for the clinical-only model and 0.000 for treat-none, representing a 57.6% relative improvement. The DCA results and nomogram visualization are presented in Figure 8.

## 4. Discussion

A 14-feature CT-based radiomics signature that can predict treatment response to first-line platinum-based chemotherapy in advanced non-small cell lung cancer was created and externally validated in this multi-center retrospective analysis. The combined radiomics–clinical model significantly outperformed the clinical-only model (external validation AUC 0.702) with strong AUC values of 0.876, 0.849, and 0.831 in training, internal validation, and external validation cohorts, respectively. The Rad-Score also demonstrated independent prognostic value for both PFS and OS, survived subgroup analysis across histological and clinical strata, and provided clinically meaningful decision-curve net benefit.

Several aspects of our findings deserve discussion. First, the dominance of texture-based features (particularly GLCM_Contrast and GLCM_Entropy) in the final panel aligns with the existing literature demonstrating that intratumoral texture heterogeneity reflects underlying biological processes (including clonal diversity, hypoxia, and inflammatory infiltration) that collectively influence chemosensitivity [36,37,38]. The univariate predictor most strongly associated with non-response in our cohort was GLCM_Entropy that measures the complexity of the spatial distribution of gray-level intensities, as reported by [39] and [40] in NSCLC radiotherapy settings. The negative correlation of Shape_Sphericity with response (OR = 0.68) is in agreement with pathophysiological knowledge, where morphologically irregular tumors—corresponding to infiltrative growth and interaction with the stroma—are less likely to respond to cytotoxic agents [41,42].

Second, the validation of our model’s external validation AUC was 0.831, which is comparable to the previous studies. The AUC for distant metastasis prediction in lung adenocarcinoma was about 0.73 reported by [43] and 0.87 reported by [44] (with no external validation). To address this important omission, we considered this study’s ability to provide rigorous external validation in a geographically different population, from a different health care system, which supports reasonable generalizability, although further validation in larger multi-ethnic cohorts is required [45,46,47]. The performance attenuation between internal and external validation (DELTA AUC = 0.018) was small and acceptable, demonstrating the ability of LASSO regularization and stability selection to avoid overfitting in the model.

Third, it is noteworthy that the final panel has features which have been decomposed by wavelets, namely Wavelet-HHH_GLCM_Homogeneity and Wavelet-LHL_FO_Mean. Wavelet features capture multi-scale textural patterns that are not present at the original image resolution and their inclusion resulted in an increase in model performance of 2.1 AUC points when compared with the model without them. It is aligned with the work of Vallières et al. [48] and Mousa et al. [49] that showed that wavelet-filtered features in soft-tissue sarcomas were useful supplementary features. Greater inter-center variation was noted for ICC values for wavelet features, however, and we recommend that future multi-center studies explicitly provide a quality indicator for the reproducibility of wavelet features. The analysis of the spatial heterogeneity (Figure 7) was qualitatively different between the responders and non-responders, which may offer visual information beyond the aggregate feature scores. Non-responding tumors had higher GLCM_Entropy and GLSZM_ZoneVariance values across the entire tumor volume [50], indicating a diffuse heterogeneity, not only necrosis. This is consistent with the biological hypothesis that resistance to chemotherapy in NSCLC is due to extensive intratumoral diversity of clones instead of the presence of small resistant clones [51,52,53]. These relationships could be further understood by integrating radiomics data with spatial transcriptomics information in the future.

Decision-curve analysis was used to calculate the net benefit of the nomogram compared to the alternative of a therapeutic threshold range, suggesting that this tool has the potential for translation [54]. The nomogram correctly identified 11.4 additional true responders per 100 patients compared with the clinical-only model, at a clinical equipoise threshold of 30% probability of response, where the benefits of chemotherapy are offset by its toxicity. This level of improvement may assist in clinical decision-making frameworks for patient stratification and trial selection. This estimate of net benefit is derived from a single retrospective cohort with one external validation site and should be interpreted as hypothesis-generating; prospective evaluation of clinical decision-making impact is required before this threshold-specific benefit can be considered actionable in routine practice.

There are a few restrictions that need to be noted. Due to the retrospective approach, selection bias is introduced, and the requirement for complete restaging imaging may have excluded patients who experienced early clinical deterioration. Although imaging protocols were harmonized to the extent possible, residual scanner-related variation in radiomic feature values may persist despite standardized preprocessing. The external validation cohort, while geographically distinct, was predominantly European ancestry; validation in East Asian and other populations is warranted, given known differences in NSCLC biology and mutation spectra [55,56]. Additionally, this study did not include patients receiving immunotherapy or targeted therapies, which now constitute first-line standard of care for PD-L1-high or driver-mutation-positive patients; the applicability of the current signature to these subgroups requires dedicated investigation (including characterization of their distinct response kinetics and outcomes, which we were unable to assess in the present chemotherapy-restricted cohort). This is particularly important because acquired resistance to EGFR-targeted therapy in NSCLC may involve noncoding RNA-mediated mechanisms, including Lnc-TMEM132D-AS1-associated osimertinib resistance and CircSETD3-mediated gefitinib resistance [54,55]. We also note that, despite ComBat harmonization and standardized preprocessing (Section 2.3), residual multi-center imaging heterogeneity cannot be fully excluded, and reproducibility of the specific feature weights and cutoffs reported here should be confirmed in additional independent cohorts before clinical adoption. Furthermore, while the model showed strong discriminative performance, this single external validation cohort, drawn from one additional center, does not capture the full breadth of scanner manufacturers, acquisition protocols, or patient populations encountered in broader clinical practice; the reported AUC and net-benefit estimates should therefore be regarded as an initial, encouraging signal of generalizability rather than definitive proof of robustness across heterogeneous real-world settings.

Future directions include prospective validation in an interventional study design; integration of PET/CT features for combined metabolic–morphological radiomics; development of deep learning-based end-to-end response prediction models; and exploration of delta-radiomics (change in features between pre- and mid-treatment scans) for adaptive treatment monitoring. Recent multimodal AI frameworks in other disease settings further support the potential value of integrating imaging, clinical, and biological data for individualized risk prediction [55,56,57].

## 5. Conclusions

This retrospective, multi-center study offers a promising, externally validated CT-based radiomics model for pre-treatment prediction of chemotherapy response in advanced NSCLC. A 14-feature signature integrating texture, shape, first-order, and wavelet features demonstrated consistent discriminative performance (AUC 0.831 in a single external validation cohort) and independent prognostic value for survival outcomes. The combined clinical–radiomic nomogram showed a clinically meaningful net benefit over standard clinical risk assessment in this dataset. These findings should be interpreted with appropriate caution given the retrospective design, the use of a single external validation cohort, and the absence of prospective validation; as such, this tool represents an early, hypothesis-generating step toward non-invasive, imaging-based precision oncology for NSCLC rather than a clinically validated decision-support instrument. Prospective, multicohort validation—ideally including additional scanner platforms, patient populations, and treatment modalities (including immunotherapy and targeted therapy)—is required before this signature can be considered for clinical implementation.

## Figures and Tables

**Table 1:** Baseline demographic and clinical characteristics across training, internal validation, and external validation cohorts.

Characteristic	Training Cohort (n = 284)	Internal Validation (n = 128)	External Validation (n = 128)
Age, median (IQR), years	63 (56–71)	65 (57–73)	62 (54–70)
Sex, Male, n (%)	172 (60.6%)	76 (59.4%)	78 (60.9%)
Sex, Female, n (%)	112 (39.4%)	52 (40.6%)	50 (39.1%)
Smoking Status			
Current/Former	198 (69.7%)	89 (69.5%)	91 (71.1%)
Never	86 (30.3%)	39 (30.5%)	37 (28.9%)
ECOG Performance Status			
0–1	226 (79.6%)	101 (78.9%)	104 (81.3%)
2	58 (20.4%)	27 (21.1%)	24 (18.7%)
Histology			
Adenocarcinoma	156 (54.9%)	69 (53.9%)	71 (55.5%)
Squamous Cell Carcinoma	93 (32.7%)	43 (33.6%)	40 (31.3%)
Large Cell/NOS	35 (12.4%)	16 (12.5%)	17 (13.2%)
TNM Stage			
Stage IIIA-IIIB	118 (41.5%)	52 (40.6%)	54 (42.2%)
Stage IVA-IVB	166 (58.5%)	76 (59.4%)	74 (57.8%)
Response (CR + PR), n (%)	136 (47.9%)	61 (47.7%)	63 (49.2%)
Non-Response (SD + PD), n (%)	148 (52.1%)	67 (52.3%)	65 (50.8%)

CR, complete response; PR, partial response; SD, stable disease; PD, progressive disease; IQR, interquartile range; ECOG, Eastern Cooperative Oncology Group; NOS, not otherwise specified.

**Table 2:** Setting for acquisition of the CT data acquisition parameters and scanner parameters throughout participating centers.

Parameter	Center A (n = 180)	Center B (n = 104)	Center C (n = 128)	Recommended Range
Scanner Manufacturer	Siemens SOMATOM	GE Revolution CT	Philips IQon Elite	-
Tube Voltage (kVp)	120	120	120–140	100–140
Effective mAs	150 (120–180)	145 (115–175)	152 (118–182)	>100
Slice Thickness (mm)	1.5	1.25	2.0	<=2.5
Reconstruction Kernel	B30f (Lung)	LUNG	B	Soft tissue
Pixel Spacing (mm)	0.68 × 0.68	0.72 × 0.72	0.70 × 0.70	<=1.0 × 1.0
Contrast Protocol	Portal venous	Portal venous	Portal venous	Yes (required)
Window Center/Width (HU)	−600/1500	−600/1500	−600/1500	−600/1500

HU, Hounsfield units; mAs, milliampere-seconds; kVp, kilovolt peak.

**Table 3:** The 14 LASSO-selected radiomic features with ICC, regression coefficients, odds ratios, and statistical significance.

Feature Name	Feature Class	ICC (Test–Retest)	LASSO Coef.	OR (95% CI)	*p*-Value
GLCM_Contrast	Texture (GLCM)	0.91	0.412	1.51 (1.28–1.78)	<0.001
GLCM_Correlation	Texture (GLCM)	0.88	−0.318	0.73 (0.61–0.87)	0.001
GLCM_Entropy	Texture (GLCM)	0.87	0.384	1.47 (1.24–1.74)	<0.001
GLRLM_LongRunEmphasis	Texture (GLRLM)	0.83	0.296	1.34 (1.14–1.58)	0.002
GLSZM_ZoneVariance	Texture (GLSZM)	0.86	0.271	1.31 (1.11–1.55)	0.004
FO_Kurtosis	First-Order Statistics	0.92	−0.243	0.78 (0.66–0.93)	0.006
FO_Skewness	First-Order Statistics	0.89	0.219	1.25 (1.06–1.47)	0.010
Shape_Sphericity	Shape Descriptor	0.94	−0.387	0.68 (0.57–0.81)	<0.001
Shape_Compactness	Shape Descriptor	0.91	−0.312	0.73 (0.62–0.87)	0.001
Wavelet-HHH_GLCM_Homogeneity	Wavelet-Filtered	0.80	0.198	1.22 (1.04–1.43)	0.018
Wavelet-LHL_FO_Mean	Wavelet-Filtered	0.82	0.176	1.19 (1.02–1.40)	0.030
GLDM_DependenceNonUniformity	Texture (GLDM)	0.85	0.163	1.18 (1.01–1.38)	0.038
FO_Energy	First-Order Statistics	0.90	0.148	1.16 (0.99–1.36)	0.064
Shape_VoxelVolume	Shape Descriptor	0.96	0.127	1.14 (0.97–1.33)	0.108

GLCM, gray-level co-occurrence matrix; GLRLM, gray-level run length matrix; GLSZM, gray-level size zone matrix; GLDM, gray-level dependence matrix; FO, first-order; OR, odds ratio; CI, confidence interval; ICC, intraclass correlation coefficient. * Bold *p*-values indicate significance at *p* < 0.05.

**Table 4:** Performance comparison between training, internal validation, and external validation cohorts for all machine learning models, radiomics-only, clinical-only, and combined radiomics–clinical models.

Model	AUC (Train)	AUC (Int. Val.)	AUC (Ext. Val.)	Sensitivity	Specificity	F1 Score
Logistic Regression	0.812	0.794	0.778	0.764	0.801	0.782
Support Vector Machine	0.841	0.819	0.802	0.791	0.823	0.807
Random Forest	0.858	0.832	0.818	0.809	0.836	0.822
Gradient Boosting (XGBoost)	0.863	0.839	0.824	0.817	0.841	0.829
Multilayer Perceptron	0.854	0.826	0.809	0.798	0.831	0.814
Radiomics-Only (LASSO-LR)	0.849	0.822	0.807	0.802	0.829	0.815
Clinical-Only Model	0.741	0.718	0.702	0.688	0.724	0.706
Radiomics + Clinical (Combined)	0.876 *	0.849 *	0.831 *	0.831 *	0.856 *	0.843 *

AUC, area under the receiver operating characteristic curve. * *p* < 0.001 compared to clinical-only model (DeLong’s method). Int. Val., internal validation; Ext. Val., external validation.

## Data Availability

Data is contained within the article.

## Abbreviations

The following abbreviations are used in this manuscript:

NSCLC	Non-Small Cell Lung Cancer
CT	Computed Tomography
AUC	Area Under the Receiver Operating Characteristic Curve
RECIST	Response Evaluation Criteria in Solid Tumors
LASSO	Least Absolute Shrinkage and Selection Operator
TRIPOD	Transparent Reporting of a Multivariable Prediction Model for Individual Prognosis or Diagnosis
IBSI	Image Biomarker Standardization Initiative
DICOM	Digital Imaging and Communications in Medicine
HU	Hounsfield Unit
ICC	Intraclass Correlation Coefficient
ECOG	Eastern Cooperative Oncology Group
IQR	Interquartile Range
CR	Complete Response
PR	Partial Response
SD	Stable Disease
PD	Progressive Disease
TNM	Tumor, Node, Metastasis
GLCM	Gray-Level Co-occurrence Matrix
GLRLM	Gray-Level Run Length Matrix
GLSZM	Gray-Level Size Zone Matrix
GLDM	Gray-Level Dependence Matrix
ROI	Region of Interest
DSC	Dice Similarity Coefficient
OR	Odds Ratio
CI	Confidence Interval
LR	Logistic Regression
SVM	Support Vector Machine
XGBoost	Extreme Gradient Boosting
NRI	Net Reclassification Improvement
IDI	Integrated Discrimination Improvement
PFS	Progression-Free Survival
OS	Overall Survival
DCA	Decision-Curve Analysis
Rad-Score	Radiomics Score
PET	Positron Emission Tomography
FDG	Fluorodeoxyglucose
PD-L1	Programmed Death-Ligand 1
MRI	Magnetic Resonance Imaging
ITK	Insight Segmentation and Registration Toolkit
mAs	Milliampere-Seconds
kVp	Kilovolt Peak
NOS	Not Otherwise Specified
HR	Hazard Ratio
DWT	Discrete Wavelet Transform
